# Serious Scrotal Hematoma Due to Injury of Inferior Epigastric Artery: A Rare Complication of Femoral Puncture

**DOI:** 10.1016/j.jaccas.2025.103281

**Published:** 2025-03-12

**Authors:** Yukihiro Uehara, Yuko Y. Inoue, Akiyuki Kotoku, Toshihiro Nakamura, Mitsuru Wada, Kengo Kusano

**Affiliations:** a Department of Cardiovascular Medicine, National Cerebral and Cardiovascular Center, Osaka, Japan; b Department of Radiology, National Cerebral and Cardiovascular Center, Osaka, Japan

**Keywords:** ablation, complication, hemorrhage

## Abstract

The inferior epigastric artery branches from the external iliac artery just above the inguinal ligament. A 50-year-old man presented with scrotal swelling after catheter ablation, and a computed tomography scan revealed a scrotal hematoma and extravasation from the inferior epigastric artery. Emergent coil embolization was performed, and the scrotal hematoma was managed conservatively. Accidental puncture of the inferior epigastric artery during femoral vein puncture may result in a scrotal hematoma because of bleeding through the inguinal canal. It is important to understand the anatomy around the inguinal region to prevent a high puncture.

## Graphical Abstract

**Figure:**
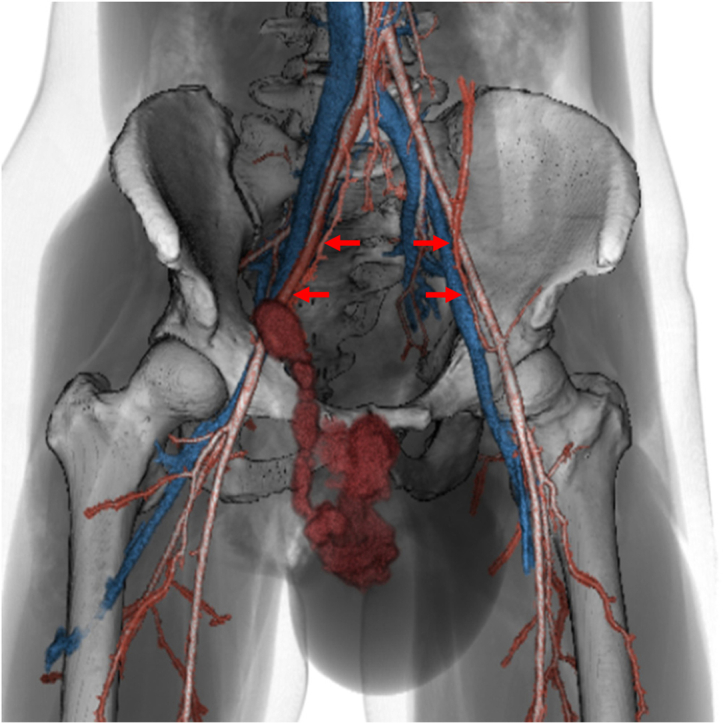

## History of Presentation

A 50-year-old man with a history of atrial septal defect surgery presented with shortness of breath on exertion, and atrial flutter was observed on a 12-lead electrocardiogram. After anticoagulation with rivaroxaban, he was considered for catheter ablation.

**Learning Objectives**

• To understand the mechanism of scrotal hematoma as a rare complication of femoral puncture.
• To learn how to avoid and manage complications of femoral puncture.

**Take-Home Messages**

• Because of a high femoral puncture site, damage to the inferior epigastric artery may occur, potentially resulting in the development of a scrotal hematoma.
• Avoiding a high puncture requires careful attention to the anatomical landmarks of the inguinal region.

## Past Medical History

**Figure 1:**
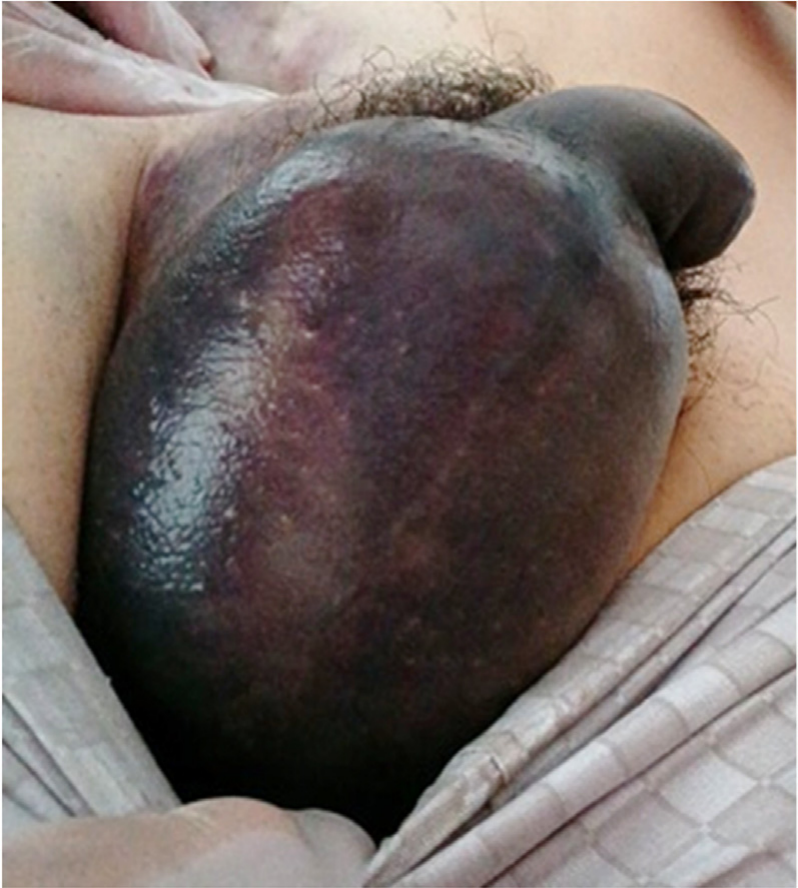
Scrotal Hematoma 1 Day After the Ablation Procedure

The right femoral vein was punctured under ultrasound guidance, and 3 sheaths were inserted for the radiofrequency ablation procedure. Heparin was administered intravenously. Common atrial flutter and incision-related atrial tachycardia were successfully terminated by catheter ablation. Manual compression and a figure-of-eight skin suture were performed during sheath removal, followed by pressure dressing for 6 hours. The day after the procedure, the patient’s hemoglobin level decreased from 15.5 to 11.9 g/dL. Upon resuming activity, he developed groin and scrotal pain that interfered with walking. Swelling at the puncture site was minimal, but significant swelling and pain were observed in the scrotum (Figure 1).

## Differential Diagnosis

Groin pain, decreased hemoglobin levels, and minimal puncture site swelling suggested the presence of a retroperitoneal and scrotal hematoma.

## Investigations

**Figure 2:**
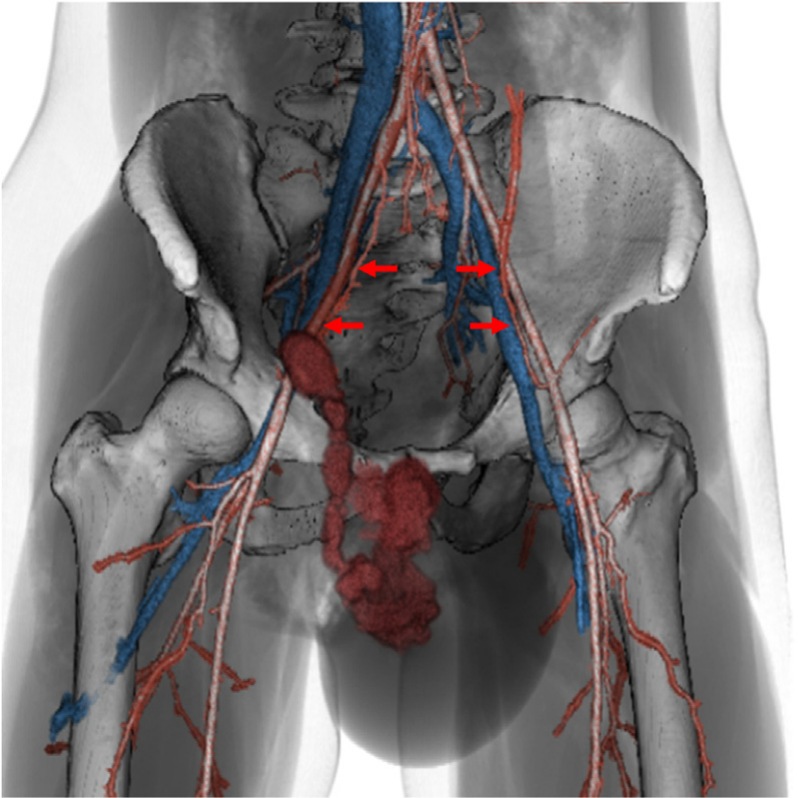
Extravasation From the Pseudoaneurysm of the Inferior Epigastric Artery With Hemorrhage Into the Scrotum The red arrows indicate the inferior epigastric arteries.

**Figure 3:**
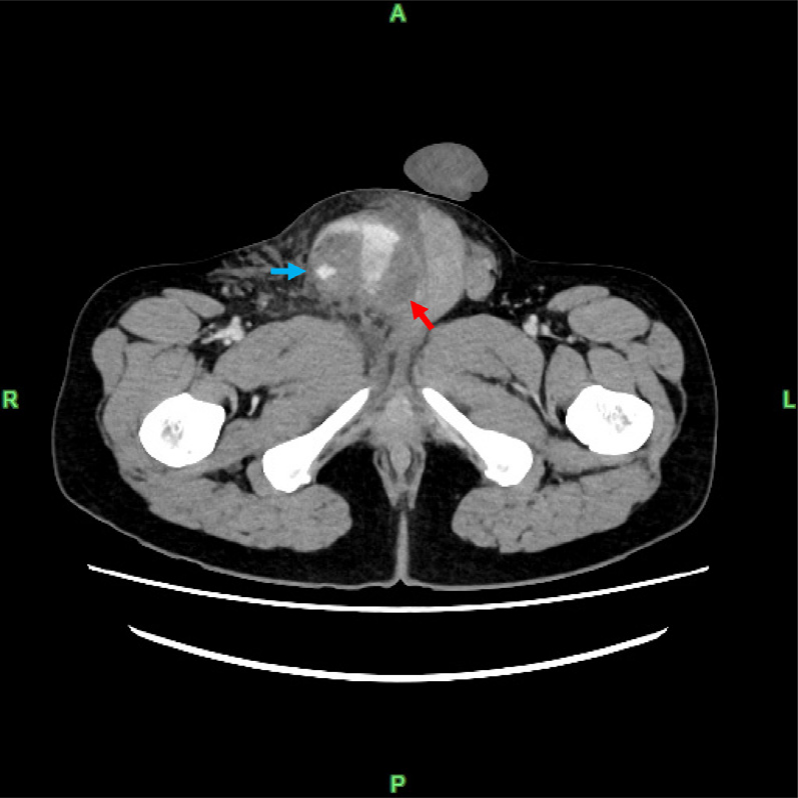
The Testis Compressed by a Scrotal Hematoma The blue arrow indicates a scrotal hematoma, and the red arrow indicates the compressed testis.

A contrast-enhanced abdominal computed tomography (CT) scan revealed contrast extravasation consistent with a pseudoaneurysm associated with an injury to the right inferior epigastric artery (IEA), a branch of the right femoral and external iliac arteries (Figure 2, Video 1). The bleeding extended through the inguinal canal into the scrotum, compressing the testes (Figure 3).

## Management

**Figure 4:**
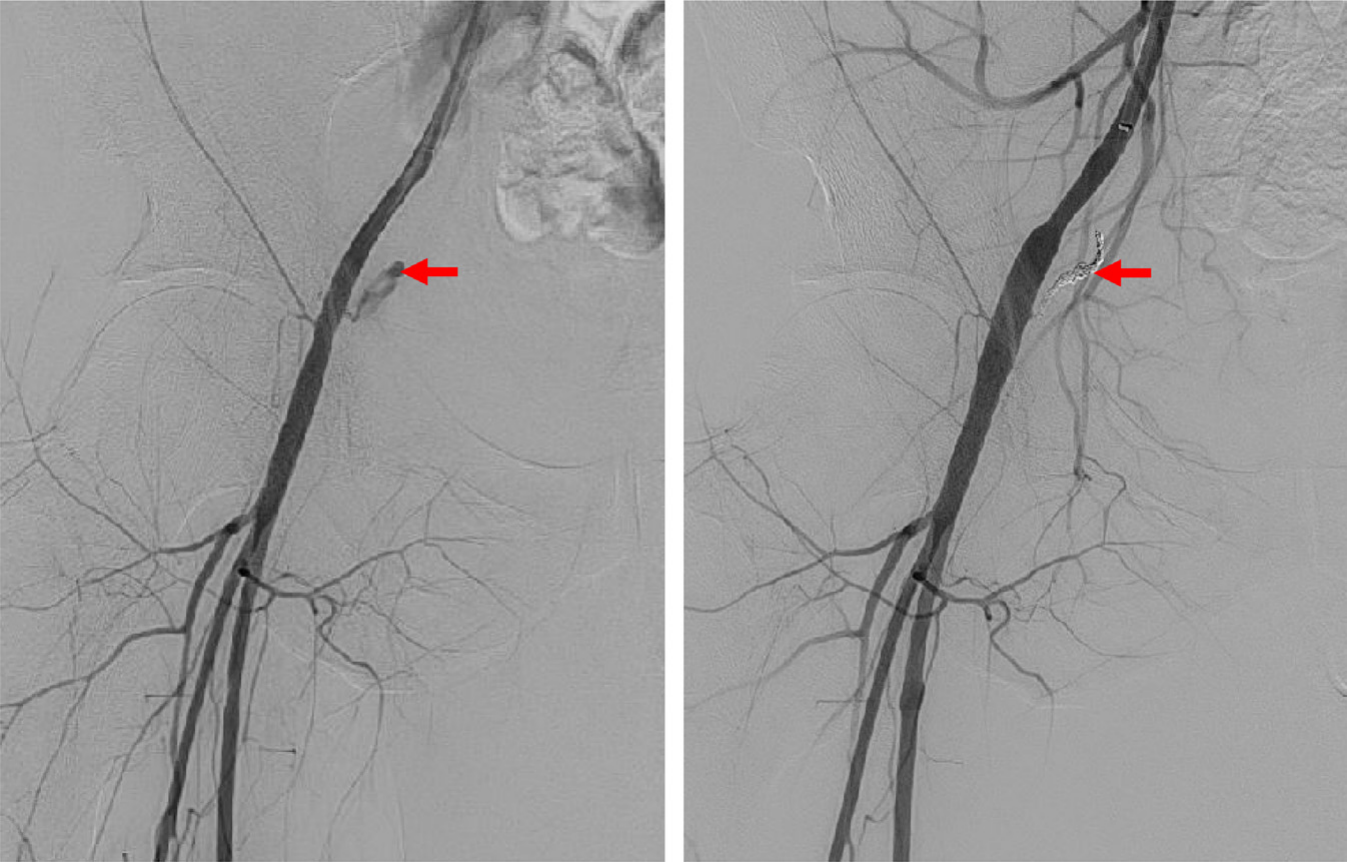
Emergent Transcatheter Coil Embolization of the Inferior Epigastric Artery Angiography revealed active extravasation (red arrow) from the right inferior epigastric artery (left panel), and hemostasis was successfully achieved with coil embolization (red arrow) (right panel).

The patient was transferred to a general hospital with a urology department, where emergency coil embolization of the right IEA was performed (Figure 4). Contrast-enhanced CT the day after embolization confirmed hemostasis. Scrotal hematoma and swelling were managed conservatively with intravenous analgesia and a urinary catheter. The hemoglobin level dropped to 5.2 g/dL the day after embolization, requiring blood transfusion, and the hemoglobin level remained stable with no further decline. After the scrotal hematoma improved, the patient was discharged home.

## Outcome and Follow-Up

At 1-month follow-up, the patient had no recurrence of arrhythmia, and scrotal swelling had significantly decreased.

## Discussion

To our knowledge, this is the first case report to demonstrate the efficacy of embolization therapy in achieving hemostasis in a patient with a large scrotal hematoma due to IEA injury associated with femoral vein puncture. Contrast-enhanced CT confirmed that blood flowed from the IEA through the inguinal canal into the scrotum, leading a large hematoma.

The IEA originates from the external iliac artery, just above the inguinal ligament, and is a major blood vessel that ascends along the abdominal wall, supplying blood to the muscles and skin of the lower abdomen. IEA injury has been reported as an iatrogenic complication of various abdominal procedures, such as abdominal wall surgical incisions, laparoscopic trocar insertion, and abdominal paracentesis in patients with coagulopathy.^1^ IEA injury is often associated with the formation of abdominal wall hematomas, and embolization is considered an effective method of hemorrhage control.^2^

Recently, 2 cases of IEA injury associated with femoral puncture, as in this case, have been reported as a cause of retroperitoneal hematoma.^3^ A previous case report also showed that IEA injury during femoral artery puncture resulted in a scrotal hematoma, and the manual compression was effective.^4^ However, in this case, embolization therapy was highly effective in achieving hemostasis for the pseudoaneurysm rupture and active bleeding.

**Figure 5:**
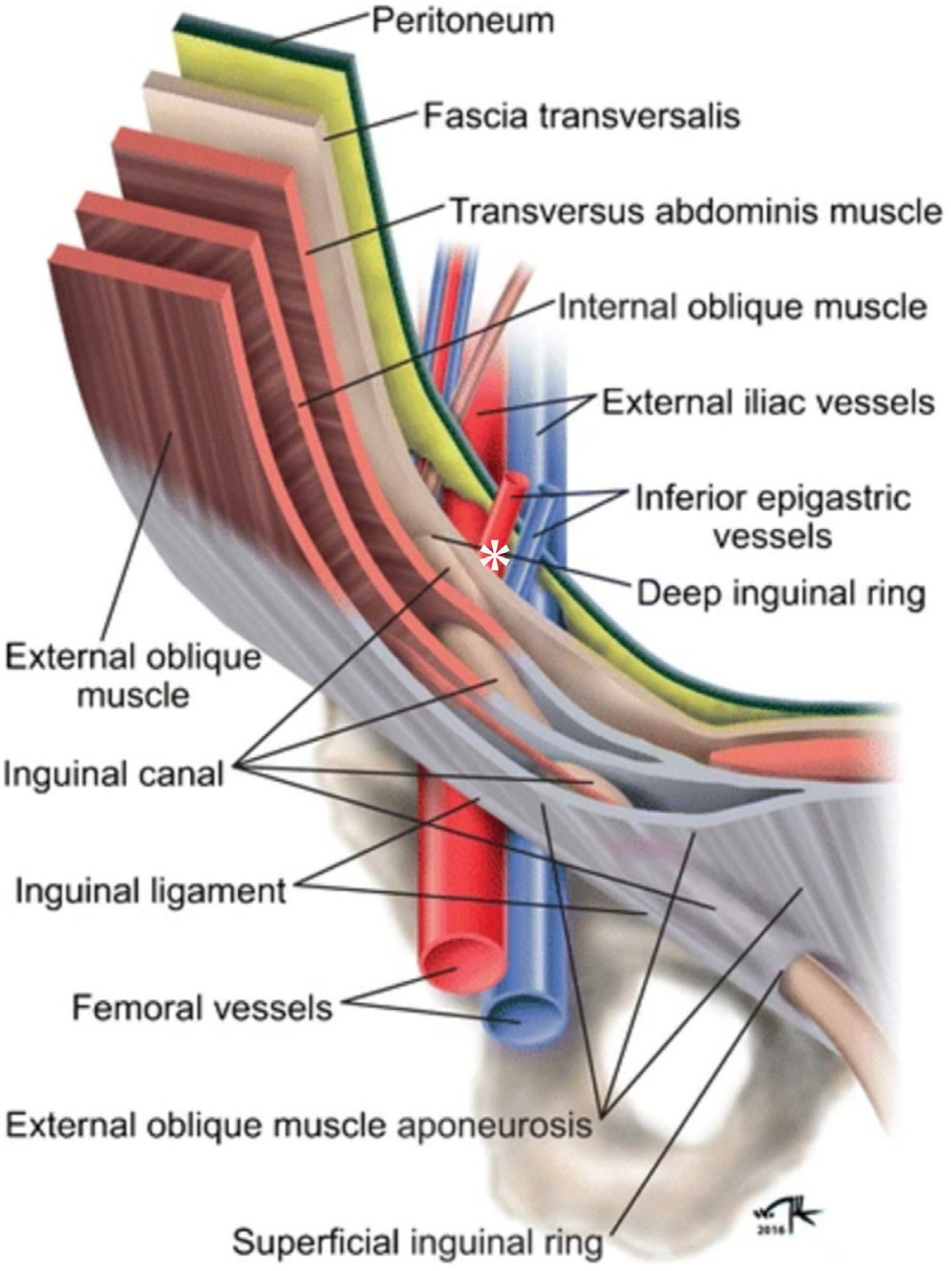
Anatomy of the Inguinal Region and the Suspected Puncture Site ∗Anatomical illustration highlights the inguinal region and suggests the puncture and bleeding of the inferior epigastric artery just above the inguinal ligament can lead to a scrotal hematoma through the inguinal canal. Reprinted from Sameshima et al.^5^

Contrast-enhanced CT demonstrated that blood flowed from the IEA into the deep inguinal ring, forming a significant scrotal hematoma through the inguinal canal. If the IEA is injured at the site immediately after its bifurcation from the external iliac artery, before it enters the abdominal muscles, a scrotal hematoma may occur as a rare but serious complication after femoral puncture (Figure 5).^5^ In this case, even though the puncture was performed under vascular ultrasound guidance, the puncture point may have been high and above the inguinal ligament. The overlap of the right femoral artery and vein distal to the inguinal ligament may have contributed to the high puncture (Supplemental Figures 1 and 2).

To prevent IEA injury, it is recommended that the puncture site be located distal to the inguinal ligament. Understanding the anatomical relationship between the iliac and pubis bones under fluoroscopic guidance and identifying arterial branches using vascular ultrasound with color Doppler can be effective preventive measures.^6^

## Conclusions

Scrotal hematoma should be considered a potential complication of femoral puncture.

## Funding Support and Author Disclosures

This work was supported by the Japan Society for the Promotion of Science (JSPS) KAKENHI (grant number JP24K10777). The authors have reported that they have no relationships relevant to the contents of this paper to disclose.

## Appendix

Video 1 Extravasation from the pseudoaneurysm of the inferior epigastric artery, and hemorrhage into the scrotum.

## References

[1] Joy P., Prithishkumar I.J., Isaac B. (2017). Clinical anatomy of the inferior epigastric artery with special relevance to invasive procedures of the anterior abdominal wall. J Minim Access Surg.

[2] Sobkin P.R., Bloom A.I., Wilson M.W. (2008). Massive abdominal wall hemorrhage from injury to the inferior epigastric artery: a retrospective review. J Vasc Interv Radiol.

[3] Fujiwara T., Ikeda H., Kuriyama A. (2022). Inferior epigastric artery injury due to femoral venipuncture for neuroendovascular intervention: two cases requiring transcatheter arterial embolization. J Neuroendovasc Ther.

[4] Aldoori J.S., Abdulfaraj A., Rasul S.M.S. (2024). Scrotal hematoma: a rare complication of transfemoral percutaneous coronary intervention. Egypt Heart J.

[5] Sameshima Y.T., Yamanari M.G.I., Silva M.A., Neto M.J.F., Funari M.B.G. (2017). The challenging sonographic inguinal canal evaluation in neonates and children: an update of differential diagnoses. Pediatr Radiol.

[6] Chun E.J. (2018). Ultrasonographic evaluation of complications related to transfemoral arterial procedures. Ultrasonography.

