# Supplementary material for: Serious Scrotal Hematoma Due to Injury of Inferior Epigastric Artery: A Rare Complication of Femoral Puncture
Source: JACC Case Rep. 2025 Mar 12;30(9):103281. doi: 10.1016/j.jaccas.2025.103281 (PMC12245472; doi:10.1016/j.jaccas.2025.103281)

Supplemental Figure 1. The anatomical relationship between the inguinal ligament and the femoral artery and vein in the present case

Red arrow: The femoral artery and vein overlap distal to the inguinal ligament. The right inguinal ligament is shown in green.


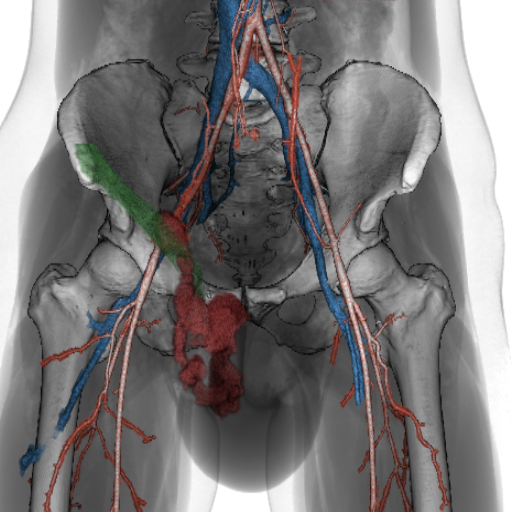


Supplemental Figure 2. Puncture site

Red arrow: Accidental puncture site of the inferior epigastric artery resulted in pseudoaneurysm and scrotal hematoma.


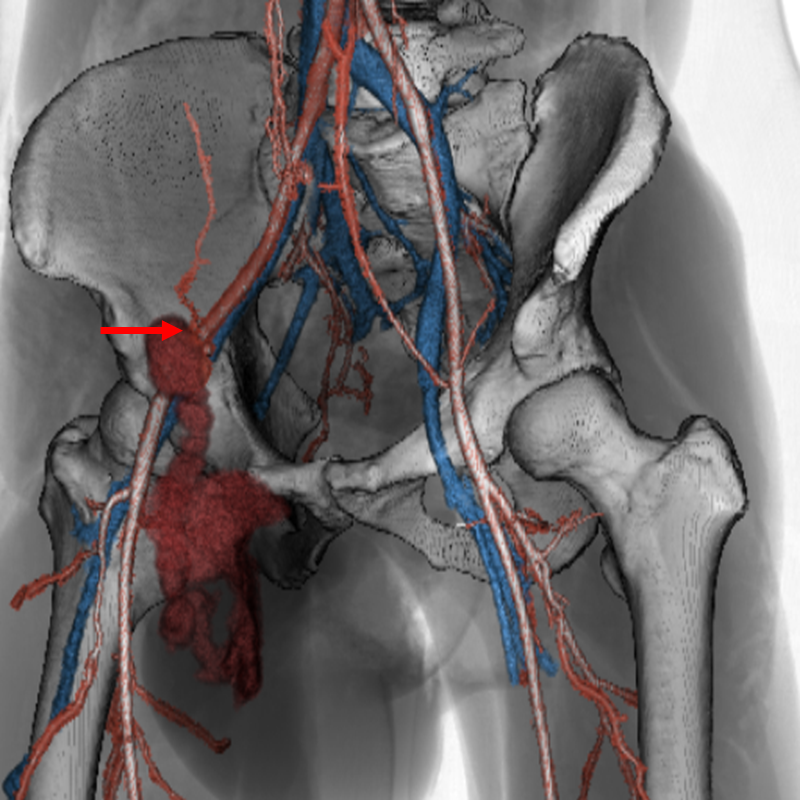

Supplement: Supplemental Figures 1 and 2 [file mmc2.docx]
